# Plant-parasitic nematode disease complexes as overlooked challenges to crop production

**DOI:** 10.3389/fpls.2024.1439951

**Published:** 2024-07-23

**Authors:** Luisa M. Parrado, Marisol Quintanilla

**Affiliations:** ^1^ Department of Plant, Soil and Microbial Sciences, Michigan State University, East Lansing, MI, United States; ^2^ Department of Entomology, Michigan State University, East Lansing, MI, United States

**Keywords:** plant-parasitic nematodes, plant diseases, disease complex, crops, interactions, management

## Abstract

Plant diseases are caused by various microorganisms such as bacteria, fungi, viruses, and nematodes. These diseases impact crop growth, reduce produce quality, and lead to financial losses. Plant disease can be caused by single pathogens or by interactions called “disease complexes”, involving two or more pathogens. In these cases, the disease severity caused by the pathogens combined is greater than the sum of the disease caused by each pathogen alone. disease complexes formed among plant-parasitic nematodes (PPNs) with bacteria, fungi, or viruses, can occur. PPNs either enhance the other pathogen incidence and severity or are necessary for disease symptoms to be expressed. PPNs can do so by being wounding agents, vectors, modifiers of plant biochemistry and physiology, or altering the rhizosphere microbiome. This review identifies several PPNs-plant pathogens disease complexes in crop production to discuss how understanding such interactions is key for improving management practices.

## 1 Introduction

Since the human population is expected to increase to 9.6 billion by 2,050 and 10.9 billion by 2,100, agriculture will need to increase by 60% to sustain such a population (Ristaino et al., 2021). However, plant pathogens represent a significant obstacle to this goal. Global yield losses due to plant pathogens are significant, ranging from 21% to 30%, and sometimes greater in certain geographic areas (Savary et al., 2019). Hence, plant diseases represent a significant threat to food and financial security and their management is crucial for human sustainability.

As molecular and statistical tools advance, so does our understanding of the complexity of the soil microbiome and its implications for plants. Therefore, the use of these tools has allowed us to conclude that co-infection of plants, both in the wild and in agriculture, is common. Pathogens can interact directly through mechanical or chemical mechanisms or indirectly through host biochemical responses. These interactions can affect pathogens’ epidemiology by shaping virulence as well as genetic diversity (Tollenaere et al., 2016). From the complex interactions within the plant microbiome, the synergistic interaction between plant pathogens can lead to disease symptoms that are greater than the sum of symptoms caused by each pathogen alone (Tamborindeguy et al., 2023). Such interactions are known as “disease complex”, thereby complicating the development of efficient management strategies (Lamichhane and Venturi, 2015).

Plant-parasitic nematodes (PPNs) and other microorganisms like fungi, bacteria, or viruses can form disease complexes (Back et al., 2002; Khan et al., 2023; Topalović and Geisen, 2023; Siddiqui and Aziz, 2024). PPNs are microscopic vermiform-like animals that possess a specialized organ called a “stylet”, which they use to puncture plant cells and absorb nutrients. PPNs significantly threaten high-value crops such as vegetables, fruits, field crops, and ornamentals and can be classified based on their feeding location and behavior, and in this review, we will focus on PPNs that feed from roots only (Kumar and Yadav, 2020; Phani et al., 2021). PPNs’ feeding behavior, secretions, the type of cell they feed from, population abundance, and time spent interacting with the plant cell define the physiological changes in the plant as well as its response. However, one of the main challenges in the identification of PPN infection is that above-ground symptoms are often mistaken as nutrient deficiency or abiotic stress (Kumar and Yadav, 2020). There are different PPN detection methods such as morphological, biochemical, PCR, isothermal amplification technology spectral techniques, and machine learning, however, all of these have disadvantages resulting in a lack of efficient and accurate identification of PPNs (Shao et al., 2023).

Additionally, the understanding of PPNs disease complexes have major limitations including determining the presence/absence of it. The use of symptom-based methodologies can be unreliable, but molecular techniques can allow us to accurately identify, quantify, and study disease complexes in depth (Tollenaere et al., 2016). This review aims to highlight the known roles of PPNs in described disease complexes to discuss how filling the knowledge gaps about the interactions of such pathogens and their effects on host plants is crucial to providing direction for the development of efficient management strategies.

## 2 Plant-parasitic nematodes disease complexes in different cropping systems

Disease complexes that involve PPNs are thought to have two types of interactions. 1. The expression of disease symptoms happens only if all pathogens are present or 2. Each pathogen causes disease, but PPNs’ presence enhances the incidence of the other pathogen. At the same time, these interactions happen depending on plant genotype, soil organic matter, nutrient content, and other microbes. Furthermore, according to recent reviews, PPNs can enhance infection by other soil-borne pathogens by either being vectors, wounding agents, or modifiers of plant biochemistry, physiology, or rhizosphere microbiome (Siddiqui et al., 2012; Ravichandra, 2014; Zhang et al., 2020). Below we will describe some cases of PPN disease complexes categorized by PPNs-bacteria, PPNs-fungi, and PPNs-virus to emphasize how the lack of current knowledge about their combined influence on plant defense response, the mechanisms of interaction, and the factors that trigger these disease complexes are key to the development of successful management tools.

### 2.1 PPNs and plant pathogenic fungi

The first evidence of PPNs-fungi interaction was recorded when the fusarium wilt of cotton was found to be more severe in the presence of *Meloidogyne* spp. by Atkinson (1892). Since then, multiple disease complexes formed between *Meloidogyne* spp. and other fungi have been described affecting several crops including tomatoes and cotton (Khan and Sharma, 2020). These disease complexes are known as Meloidogyne-based disease complex (MDC), in which different species such as *M. incognita* and *M. javanica* associate with different fungi like *Fusarium* spp, *Rhizoctonia* spp., *Sclerotium* spp., *Phytophthora* spp., *Pythium* spp., *Ralstonia* spp., and *Alternaria* spp., affecting multiple crops (Archana et al., 2023). Moreover, the fact that *Meloidogyne* species cause physiological changes in the host such as inducing the formation of giant cells rich in nutrients, may suggest that these serve as substrates for rotting and wilt-causing fungi to proliferate, increasing plant disease severity (Siddiqui and Aziz, 2024). In addition, it is suggested PPN infection stimulate pathogenic fungi infection through root exudates. In a recent study, Lamelas et al. (2020) determined that in coffee and tomato, the MDC process is mediated by the metabolism of associated bacteria communities. They found that rather than being associated with a specific microbiome profile, MDC infection is linked to specific metabolic profile shifts that may ensure infection success like the presence of bacteria that can metabolize phenolic compounds, a plant defense response to limit *Meloidogyne* proliferation within roots. Therefore, such results then suggest that the study of PPNs disease complexes should take into consideration the effect of the plant microbiome, which may, indirectly, increase the success odds of the disease complex.

Most described PPNs and fungal disease complexes occur between widespread pathogens. For instance, *Meloidogyne* spp. and *Pratylenchus* spp., which can infect hundreds of different plant species under different environmental conditions, often form disease complexes with fungal pathogens like *Fusarium* spp., *Rhizoctonia* spp., *Phytophthora* spp., and *Verticillium* spp. (Zhang et al., 2020). Laasli et al. (2022) found that when inoculating 150 spring wheat lines with *Pratylenchus thornei* and the crown rot fungus *F. culmorus*, only 48 were resistant to *P. thornei*, while 16 lines were moderately resistant to *F. culmorus*. Coinoculation caused a downgrade of resistance in the wheat lines, increasing disease severity even more when the fungus was inoculated first. However, other literature shows that the relationship between some PPNs and pathogenic fungi can be antagonistic. For example, Ahmadi et al. (2022) found that dryland crown rot *F. culmorus* caused a reduction of *Heterodera filipjevi* numbers and the disease was more severe in plants under drought. The authors then speculate that the early senescence and death of the host caused by coinfection and environmental conditions, may disrupt the nematode cycle, reducing its numbers. Similarly, Hassan et al. (2012) found that inoculating wheat plants with *H. avenae* one month before *F. culmorus*, showed a synergistic interaction, reducing yield by 44%. Meanwhile, inoculating *F. culmorus* first, resulted in a yield reduction of 33% and nematode numbers decreased. Likewise, it was demonstrated that *H. glycines* and *P. sojae* can cause more severe disease in soybeans (Chowdhury et al., 2022). Interestingly though, nematode numbers were reduced when soybean plants were inoculated with *P. sojae* and they speculate that because both pathogens share the same niche, the fungus can decrease availability of nutrients as well as create a hostile environment for the nematode to complete its life cycle. Hence, the presence of the pathogens may not be the only indicator of a disease complex, but the timing of separate infections, the implications of coinfection to each pathogen, host susceptibility and the niche conditions need to be conducive to demonstrate a synergistic interaction between pathogens.

### 2.2 PPNs and plant pathogenic bacteria

Through the feeding process, PPNs create open wounds and modify plant biochemistry and physiology which make the plant susceptible to bacterial infection (Siddiqui et al., 2012; Archana et al., 2023; Khan et al., 2023). The first possible interaction between nematodes and bacteria was observed when tomatoes planted in nematode-infested soil were infected with *Pseudomonas solanacearum* and tomatoes remained bacteria-free in nematode-free soil (Hunger, 1901). Later, Lucas et al. (1955) used a tobacco variety resistant to *P. solanacearum* and found that when co-inoculated with *M. incognita*, the tobacco plants were infected with *P. solanacearum* and expressed bacterial wilt symptoms. Similarly, it was reported that the presence of *Meloidogyne* spp. can break down the resistance of tobacco to *Ralstonia* spp. and *Xanthomonas* spp (Johnson et al., 2005). This indicates that while PPNs provide open courts of infection for bacteria, PPNs infection can also interfere with plant defense responses. Therefore, transcriptomic studies that focus on the effect of PPNs on plant gene expression related to defense mechanisms can provide a deeper understanding of how nematodes break plant resistance (Li et al., 2023).

In another case, chili co-inoculations of *M. javanica* and *R. solanacearum* at different densities were determined and the authors found that the incidence of *R. solanacearum* wilt was the lowest when *M. javanica* was absent while when both pathogens were co-inoculated at the highest density disease severity was the highest (Asghar et al., 2020). Similarly, potatoes can be affected by *Meloidogyne* spp. and *R. solanacearum* separately, but when co-inoculated, it was found that *M. incognita* disease incidence and yield loss were greater (Archana et al., 2023). On eggplant, co-inoculation of *M. incognita* with *R. solanacearum* and *Phomosis vexans* showed a significant decrease in plant growth, chlorophyll content, and carotenoid percent, however, the decrease in plant growth was the greatest when the nematode was inoculated 20 days before (Khan and Siddiqui, 2017). A recent study by Topalović et al. (2022) revealed the PPN associated bacteria in *M. hapla* conducive and suppressive soils. By integrating controlled greenhouse experiments with amplicon sequencing with technologies like Illumina MiSeq and bioinformatics, the results suggest that some of the bacteria attached to the nematode cuticle potentially protects and aid the nematode parasitism process. Nevertheless, overall, the studies of disease complexes between pathogenic bacteria and PPNs are not as extensive as those with pathogenic fungi, indicating a need for vigorous experiments like the one mentioned above.

### 2.3 PPNs and plant pathogenic viruses

PPNs mainly act as vectors of pathogenic viruses. The first report of PPNs vectoring plant viruses was by Hewitt et al. (1958) when they observed *grapevine fanleaf virus* transmission via the dagger nematode *Xiphinema index*. The only two families of ectoparasitic PPNs that have been proven to be vectors of plant viruses are the Longidoridae, which transmit nepoviruses, and the Trichodoridae, which transmit tobaviruses (Sarwar et al., 2020; Singh et al., 2020). Briefly, these nematodes acquire and transmit the viruses through feeding with their stylet, while retaining the virus in specific sites in their esophagus (Sarwar et al., 2020; Singh et al., 2020).

PPN transmission of a virus starts with ingestion, followed by acquisition, adsorption, retention, release, transmission and establishment (Singh et al., 2020). For the latter to be successful, host plant species influence successful virus transmission. For example, Demangeat et al. (2004) found that while *X. index* can acquire *grapevine fanleaf virus* from grapevine and quinoa plants, the virus can only be transmitted to grapevine. In addition, relationship specificity between virus and PPNs is determined by the virus coat protein and the ability of the nematode to retain virus particles (Brown and Weischer, 1998; Andret-Link et al., 2004; Sarwar et al., 2020). Moreover, their synergistic interaction can be specific, or through effects of the virus on the host plant, that may affect PPNs (Sarwar et al., 2020). Although it is clear how the virus benefits from the nematode, how the nematode benefit from the virus remains unknown.

## 3 Future directions: filling knowledge gaps for the development of efficient management tools for PPNs disease complexes

PPNs disease complexes can be specific as well as influenced by biotic and abiotic factors. Synergistic interactions may be specific as in the interaction may vary depending on the pathogen genotype, nematode species and ultimately, their effect on the host plant. As an example, on potatoes, the interaction between *Verticillium dahliae* and *Pratylenchus* species was only enhanced with *P. penetrans*, however, variations on gene fragments within species may explain why no synergistic interaction with *V. dahliae* is observed with *P. neglectus* populations (Riedel et al., 1985; Hafez et al., 1999). Likewise, there are multiple vegetative compatibility groups of *V. dahliae* but VCG4A is more aggressive and caused more severe disease in *P. penetrans* infested soil, while in peppermint and spearmint, there was a stronger interaction with VCG4B (Botseas and Rowe, 1994; Johnson and Santo, 2001).

As for abiotic factors, it was found that co-inoculation of a bean genotype resistant to both *F. oxysporum* and *M. incognita* led to wilt symptoms only at 27°C (France and Abawi, 1994). In another study, heavier soils were more conducive to chickpea infection by *F. oxysporum*, while loamy soils were more suitable for *M. javanica* infection (Maheswari et al., 1997). In addition to soil type and temperature, PPNs infection can alter root exudates composition, which can increase the attraction of other pathogens. For instance, Van Gundy et al. (1977) found that within 14 days of infection with *M. incognita* root exudates were high in carbohydrates, while after 14 days, nitrogenous compounds increased. Thus, a shift in C:N ratio was associated with *R. solani* infection. Lastly, soil pH can influence the parasitism success of PPNs. Chen et al. (2012) found that *Pratylenchus* sp. and *Xiphinema* sp. correlate negatively with soil sand content and pH, respectively. Therefore, abiotic factors are significant drivers of PPNs disease complexes and should be taken into consideration when validating synergistic interactions (Back et al., 2002).

Aside from accurate experimental design, the selection of statistical tools is critical. For instance, to determine at which thresholds pathogens interactions are triggered, the significance of pathogen genotypes, host genotype, and inoculation times, the use of multifactorial analysis tools could provide a better insight (Trivedi et al., 2022; Shoaib et al., 2023). For example, Wheeler et al. (2019) combined field experiments and advanced statistical tools such as multiple linear regression, generalized additive model, random forest, and artificial neural network to conclude that while *V. dahliae* was thought to be the primary predictor of mint wilt, all models selected *Pratylenchus* spp. as the most important predictor. This emphasizes how underestimated the contributions of PPNs are in disease complexes, and how important it is to integrate different tools of data analysis to maximize the understanding of drivers of plant disease and avoid contradictions among disease complexes reports. Good experimental design and data collection together with statistical tools like multivariate and non-parametric analysis can provide accurate answers regarding disease dynamics, while simulation models can identify the main drivers of disease and predict it, becoming a practical tool for management decisions (Nayak et al., 2018).

Likewise, with the rapid advance and accessibility of genomic tools, it is necessary to dive deeper into the study of the expression of genes related to virulence and pathogenicity when the pathogens are interacting, and the effect that the pathogens combined have on the host expression of genes related to plant defense, physiology, and fitness (Rocha and Schwan, 2023). Results from such research can help to identify targets for the development of new chemistries as well as improve plant breeding of resistant varieties (Eves-van den Akker, 2021). In conclusion, considering the current knowledge of PPNs disease complexes, the applications of thorough statistical tools, and the availability of genomic resources and tools, it is indispensable to adapt these advanced technologies when developing research ideas for grant proposals and planning experiments. The knowledge generated from experiments that take into consideration what was discussed in this review may positively impact the development of new chemistries for management of PPNs disease complexes, improve plant breeding and resistance mining, and ensure timely and effective pest management decisions.

## Author contributions

LP: Writing – original draft, Writing – review & editing. MQ: Writing – review & editing.
